# Selective up-regulation of NMDA-NR1 receptor expression in myenteric plexus after TNBS induced colitis in rats

**DOI:** 10.1186/1744-8069-2-3

**Published:** 2006-01-17

**Authors:** QiQi Zhou, Robert M Caudle, Donald D Price, Arseima Y Del Valle-Pinero, G Nicholas Verne

**Affiliations:** 1 Department of Medicine, University of Florida College of Medicine, Gainesville, FL 32610, USA; 2 Department of Oral and Maxillofacial Surgery, University of Florida College of Dentistry, Gainesville, FL 32610, USA; 3 Department of Neuroscience, University of Florida College of Dentistry, Gainesville, FL 32610, USA; 4 North Florida/South Georgia VA Health System, USA

## Abstract

**Background:**

N-methyl-D-aspartic acid (NMDA) spinal cord receptors play an important role in the development of hyperalgesia following inflammation. It is unclear, however, if changes in NMDA subunit receptor gene expression in the colonic myenteric plexus are associated with colonic inflammation. We investigated regulation of NMDA-NR1 receptor gene expression in TNBS induced colitis in rats. Male Sprague-Dawley rats (150 g–250 g) were treated with 20 mg trinitrobenzene sulfonic acid (TNBS) diluted in 50% ethanol. The agents were delivered with a 24 gauge catheter inserted into the lumen of the colon. The animals were sacrificed at 2, 7, 14, 21, and 28 days after induction of the colitis, their descending colon was retrieved for reverse transcription-polymerase chain reaction; a subset of animals' distal colon was used for two-dimensional (2-D) western analysis and immunocytochemistry.

**Results:**

NR1-exon 5 (N1) and NR1-exon 21 (C1) appeared 14, 21 and 28 days after TNBS treatment. NR1 pan mRNA was up-regulated at 14, 21, and 28 days. The NR1-exon 22 (C2) mRNA did not show significant changes. Using 2-D western analysis, untreated control rats were found to express only NR1_001 _whereas TNBS treated rats expressed NR1_001_, NR1_011, _and NR1_111_. Immunocytochemistry demonstrated NR1-N1 and NR1-C1 to be present in the myenteric plexus of TNBS treated rats.

**Conclusion:**

These results suggest a role for colonic myenteric plexus NMDA receptors in the development of neuronal plasticity and visceral hypersensitivity in the colon. Up-regulation of NMDA receptor subunits may reflect part of the basis for chronic visceral hypersensitivity in conditions such as post-infectious irritable bowel syndrome.

## Background

Visceral pain is a common symptom involved in many gastrointestinal disorders such as inflammatory bowel disease and irritable bowel syndrome. During the last decade research focusing primarily on alterations in the peripheral and central nervous system has improved our understanding of the pathophysiological mechanisms of chronic visceral pain. These studies have demonstrated significant physiological changes following injury to the viscera in the firing patterns of both primary afferent neurons that transmit nociceptive information from the viscera and in central neurons that process the nociceptive information [1-7]. Furthermore, a number of receptors, neurotransmitters, cytokines and second messenger systems in these neurons have been implicated in the enhancement of visceral nociception [8-12]. Previous research in the enteric nervous system has focused primarily on altered motility. The potential role of altered enteric nervous system function on visceral nociception has not been fully explored. In this study, we examined potential mechanisms of visceral pain produced by colitis. We used an in vivo inflammatory model of TNBS colitis which revealed N-Methyl-D-aspartate (NMDA) receptors modulated neuronal plasticity.

In the spinal cord, NMDA receptors were found to play a pivotal role in the development and maintenance of allodynia and hyperalgesia in both visceral and somatic tissue [13-18]. NMDA receptors integrate the activity of groups of neurons and provide a mechanism to amplify nociceptive signals. This process leads to central sensitization, which is characterized by enlarged neuronal receptive fields, allodynia and hyperalgesia [7,16,18-25]. Recent work demonstrated the presence of NMDA receptors in the enteric nervous system [22,26-29]. The role of these receptors is currently not known, but it is likely that they serve to integrate and amplify signals within the network, possibly resulting in altered gut motility, secretion, and enhanced nociception.

NMDA receptors are composed of at least two subunits, NR1 and NR2 [30-32]. A third subunit of the NMDA receptor, NR3, has also been described, but it is not required for a functional receptor and its role is currently unclear [33,34]. The NR1 subunit forms eight functional splice variants based on the presence or absence of three alternatively spliced exons, Exon 5 (N1), Exon 21 (C1) and Exon 22 (C2) [35-39]. The presence of N1 enhances the current flow through the NMDA receptors and prevents glycine independent stimulation of the receptors by spermine [40,41]. The C1 cassette contains four serines that are known phosphorylation sites and an ER retention signal. Phosphorylation of the serines blocks the ER retention signal and allows transport of the receptors to the plasma membrane [42-44]. The presence of the C2 cassette alters the C-terminus of the protein and changes the targeting of the protein for different cell structures [39,45]. Thus, the various splice variants of NR1 have distinct properties that significantly influence the function of the fully formed receptor. In this current study, we examined the expression of the NR1 splice variants in the colon of rats following TNBS treatment to determine if NMDA receptor function was altered by an inflammatory injury. We hypothesized that there may be enteric nervous system sensitization mediated by increased expression of NR1 splice variants following an inflammatory injury to the gut by TNBS colitis. The resulting sensitization of the colonic myenteric plexus would be similar to other chronic disorders where peripheral sensitization is present.

## Materials and methods

### Animal preparation

Male adult Sprague-Dawley rats weighing 150–250 g were used in this experiment. The rats were housed in pairs under constant temperature and humidity with 12-hour light-dark cycles, and were given free access to food and water. Administration of intracolonic trinitrobenzene sulphonic acid (TNBS) with 50% ethanol was used to produce colonic ulceration and inflammation [46]. Prior to instillation of TNBS in the colon, the animals were anesthetized with an intraperitoneal injection of sodium pentobarbital (50–90 mg/kg). Following this, 20 mg (per rat) of trinitrobenzene sulfonic acid (TNBS) (1 M in Water, Sigma Chemical Co.) diluted in 50% ethanol, was instilled into the lumen of the colon via the anus (n = 58: RT-PCR n = 3 per time point; 2-D western analysis n = 1 per treatment; immunohistocytochemistry n = 2 per treatment; colorectal distension testing n = 8 per time point). The agent was delivered in a volume of 0.3 ml/rat with a 24 gauge catheter. An equivalent volume of saline was injected into control rats (n = 8: RT-PCR n = 3; colorectal distension testing n = 5).

### Evaluation of colonic inflammation

Immediately following the somatic and visceral pain testing, all rats were euthanized using sodium pentobarbital (120 mg/kg, ip). Following euthanasia, 3 cm of the descending colon was removed and processed for histopathology. The tissue was fixed in formalin and processed using standard techniques for H & E staining. The severity of the lesions in the colon and mucosa was graded using a system previously described by Al Chaer et al [1]. The grades of colitis included: mild (+1) infiltration of a limited number of neutrophils in the lamina propria with minimal interstitial edema; moderate (+2) infiltration of a moderate number of neutrophils in the lamina propria with moderate interstitial edema; severe (+3) diffuse infiltration of neutrophils in the lamina propria with severe interstitial edema.

### Colorectal distension testing

A subset of treated and saline-treated rats underwent colorectal distension. These rats were not used for any of the molecular marker studies. A 3 cm long balloon was used to perform colon distension. The balloon was lubricated and placed into the rat's distal colon so that the tip of the balloon was 1 cm from the anus. The rats were allowed 10 minutes to acclimatize before behavioral testing began. Using an automated distension device (G & J Electronic Inc. Toronto, Canada) the rats received phasic distension (0–80 mmHg in 5 mmHg ascending increments) of the colon until the first contraction of the testicles, tail, or abdominal musculature occurred which was indicative of the first nociceptive response as previously described [47]. The colonic distensions were repeated 4 times and the mean pressures at the nociceptive threshold were recorded for each rat.

Rats were euthanized with sodium pentobarbital (120 mg/kg, ip). Immediately following euthanasia the descending colon (~2–3 cm) was removed at 2, 7, 14, 21, and 28 days following TNBS treatment. Untreated rats (n = 6) and saline rats (n = 3) were both used as controls. Tissue was prepared for RNA and protein extraction for 2-D western analysis, as well as immunocytochemistry.

All procedures were approved by the North Florida/South Georgia Veterans Health System Institutional Animal Care & Use Committee

### Reverse transcription polymerase chain reaction

All primers of NR1 subunits for RT-PCR were synthesized (GenoMechanix, Gainesville, FL). The NR1 receptor subunit-specific primers were designed by targeting three alternatively spliced exons (Table 1) [48]. Glyceraldehyde phosphate dehydrogenase (GAPDH) was used as an internal control since its expression is not regulated by inflammation [49]. RNA was extracted using the RNeasy Mini Kit (QIAGEN Inc). RT-PCR was carried out following the manufacturer's instructions by using the RT-PCR Access kit (Promega Corporation). The temperature cycle (Eppendorf-Master Cycler Gradient from Brinkmann Instruments Inc) followed the guidelines provided by Promega Corporation. Reverse transcription (RT) period was 48°C/45 min (RT time), 94°C/2 min (initial denaturing of RT), 72°C/1 min (extension of RT time). The temperature cycle of PCR was 94°C/30 min (denaturing), 50–62°C/1 min (annealing), 72°C/2 min and 72°C/7 min (extension). A total of 25–30 cycles were conducted. The PCR product samples were loaded in parallel with a 100 bp DNA ladder (BIO RAD Inc) on 1.2 % ethidiumibromde-stained agarose gel. The gels were imaged with the AlphaEaseFC program using the FluorChem™ 8900. Scion Image program was used to analyze the data for statistical analysis.

**Table 1:** PCR Amplification Primers

**Primer name**	**Sense/Antisense**	**Primer Sequences**	**Product**
NR1 Pan	Sense	5'-CCCTCAGACAAGTTCATCTACGC-3'	563 bp
	Antisense	5'-AGGTTCTTCCTCCACACGTTCAC-3	
NR1Exon5 minus/	Sense	5'-GCGAGTCTACAACTGGAACCAC-3'	210 bp
NR1Exon5 plus	Antisense	5'-CTCGCTTGCAGAAAGGATGATG-3'	273 bp
NR1 Exon 21^48^	Sense	5'-TGTGTCCCTGTCCATACTCAAG-3'	307 bp
	Antisense	5'-GTCGGGCTCTGCTCTACCAC-3'	
NR1 Exon 22	Sense	5'-CATGGCAGGGGTCTTCATGCTG-3'	362 bp
	Antisense	5'-GAACACAGCTGCAGCTGGCCCT-3'	
GAPDH	Sense	5'-CCTTCATTGACCTCAACTACATGGTCTA-3'	720 bp
	Antisense	5'-TAGCCCAGGATGCCCTTT-3'	

### Two-dimensional polyacrylamide gel electrophoresis

Two-dimensional (2-D) electrophoresis was performed according to the methods of O'Farrell [50] as follows (Kendrick Labs): Isoelectric focusing was carried out in a glass tube with an inner diameter of 2.0 mm pH 3.5–10 (Amersham Biosciences, Piscataway, NJ) for 9600 volt-hrs. One μg of an IEF internal standard, tropomyosin, was added to the sample. The protein migrates as a doublet with lower polypeptide spot of MW 33,000 and pI 5.2. Following equilibration for 10 minutes in 'O' buffer (10% glycerol, 50 mM dithiothreitol, 2.3% SDS and 0.0625 M tris, pH 6.8), the tube gel was sealed to the top of a stacking gel that overlaid a 10% acrylamide slab gel (0.75 mm thick). SDS slab gel electrophoresis was carried out for about 4 hrs at12.5 mA/gel. After slab gel electrophoresis the gel was placed in transfer buffer (12.5 mM Tris, pH 8.8, 8.6 mM Glycine, 10% MeOH) and transblotted onto a PVDF membrane overnight at 200 mA and approximately 100 volts/two gels. The following proteins (Sigma Chemical Co.) were added as molecular weight standards to a well in the agarose that sealed the tube gel to the slab gel: myosin (220,00), phosphorylase A (94,000), catalase (60,000), actin (43,000) carbonic anhydrase (29,000) and lysozyme (14,000). One membrane from each group was used throughout the entire 2-D experiment. Membranes from control rats and TNBS treated rats after 14 days were used. The western blots were viewed using enhanced Chemiluminescent detection and radiographic film. Primary antibodies were removed with western blot stripping buffer (Pierce Co.) for multiple antibody probings. The efficiency of the stripping procedure was verified by using the secondary antibody and re-exposing the membrane to film.

### Immunoflorescence

Rats were euthanized with sodium pentobarbital (120 mg/kg, ip) and their descending colon (~2–3 cm) was removed at 14 days in both controls and those treated with TNBS. The tissues were put into cold saline and frozen in super-cooled isopentane. Five-micron-thick sections of descending colon were cut longitudinally in a cryostat at -20°C. Every fourth to fifth section was collected and serially mounted on a glass slide and air-dried for 1–3 hours. Tissues were placed in a blocking buffer containing 3% Normal Goat Serum (NGS) or 2% of Bovine Serum Albumin (BSA, Sigma) with PBS for 30–60 minutes, then incubated with anti-NR1pan antibody (BD Biosciences), anti-NR1-C1 antibody (courtesy of Caudle laboratory) and NR1-N1 antibodies (courtesy of Caudle laboratory) in 3% of NGS/PBS overnight at 4°C. After 3–4 washes the tissue was placed in PBS (15 minutes each) and 60-minutes in a secondary IgG (Molecular Probes, Inc.) with 3% goat serum in PBS. The NR1 pan was probed by using Alex fluor 488 (Molecular Probes, Inc.) with FITC broadband filter. The NR1-C1 and NR1-N1 were probed by using Alex fluor 594 (Molecular Probes, Inc.) with Texas Red broadband filter (Zeiss Oberkochen, Germany). The sections were washed with distilled water before mounting the section. Double-labeled sections were coded, viewed on an Axio-phot immuno-fluorescence microscope (Zeiss Oberkochen, Germany), and photographs were made.

### Data analysis

The RT-PCR data and the colorectal distension data were analyzed using ANOVA followed by Dunnett's post-test comparisons. GraphPad Prism software was used in all data analysis. A p value of <0.05 was considered significant.

## Results

### Histology

All rats treated with TNBS had severe (+3) colitis characterized by diffuse infiltration of neutrophils in the lamina propia with severe institial edema. The severe colitis was present at all time points (2, 7, 14, 21, and 28 days) following TNBS treatment.

### Colorectal distension testing

**Figure 1:**
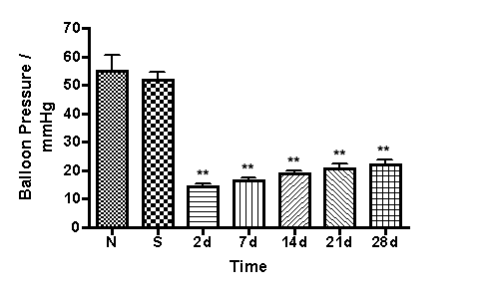
Bar graph of colorectal distension in mmHg vs. days following TNBS treatment. Normal control (N). Saline control (S). The asterisks indicated Significant differences from respective controls. One way ANOVA (p < 0.0001) with Dunnett's multiple comparison test. ** p < 0.01. Values are expressed as Means ± SEM.

Hypersensitivity to colon distension was increased in TNBS treated rats (n = 8 per time point) at 2, 7, 14, 21, and 28 days following TNBS administration compared to saline treated rats (n = 5) and control rats (n = 5) One-way analysis of variance indicated p < 0.0001 (Figure 1). Dunnett's post-test revealed that the p value was < 0.001 at 2, 7, 14, 21, and 28 days after TNBS treatment when compared to saline controls.

### RT-PCR

Primers of NR1 pan, NR1-Exon 21(C1), NR1-Exon 22 (C2) and NR1-Exon 5 (N1) were used to monitor NMDA receptor subunit mRNA expression following TNBS colitis.

**Figure 2:**
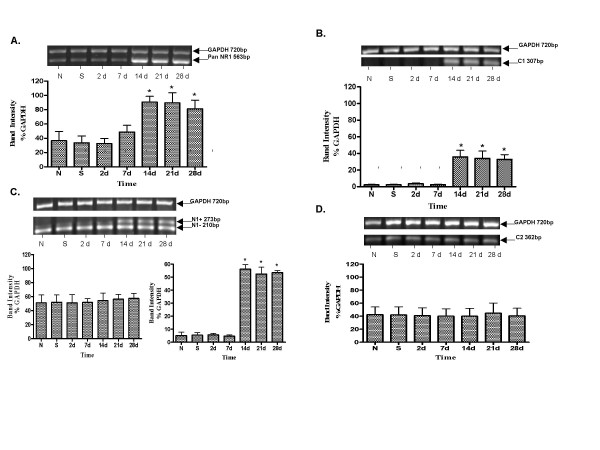
(A-D) RT-PCR analysis of changes in NMDA NR1splice variants in rat descending colon following TNBS induced colitis. Examples of agarose gel electrophoresis of PCR products are shown on the top of each panel. The RT-PCR products with specific size are indicated. The samples from naïve animals are shown in "N", saline control animals are shown in "S". GAPDH PCR was used as an internal Control. The bottom of each panel shows the summary of the effects on NMDA NR1 receptor splice variant mRNA expression. Each value represents mean of three Individual experiments. The asterisks indicated significant differences from respective controls (One ANOVA with Dunnett's post-test comparison test); p < 0.05. Statistical comparisons were made among all groups using raw data. The mRNA expression of NR1 subunit normalized to GAPDH expression are present as the Means ± SEM. A. Expression of NR1 pan mRNA increase at day 14, 21 and 28 following colitis B. Expression of NR1 C1 cassette mRNA appeared at day 14, 21 and 28 after colitis C. Expression of NR1 N1 cassette of N1 plus mRNA appeared at day 14, 21 and 28 days after colitis; expression of NR1 N1 minus was not regulated by TNBS induced colitis D. Expression of NR1 C2 cassette mRNA was not regulated by TNBS induced colitis

The RT-PCR probe that recognized all splice variants (NR1 pan) demonstrated a significant increase in NR1 expression (ANOVA F_6,14_= 6.28, p = 0.0022) following TNBS injection. Figure 2A demonstrates an increase in NR1 mRNA at 14 (p < 0.05), 21 (p < 0.05) and 28 days (p < 0.05).

The NR1-C1 splice variant mRNA was not detected in untreated and saline control rats, or 2 and 7 days following TNBS treatment. The NR1-C1 receptor mRNA appeared at 14 (p < 0.01), 21 (p < 0.01) and 28 days (p < 0.01) following TNBS injection. ANOVA results showed F_6,14 _= 11.46, p < 0.0001 (Fig 2B). The NR1-N1 minus splice variant mRNA is not regulated by TNBS induced colonic inflammation (ANOVA p = 0.99). The mRNA expression was constant throughout the observation period. However, NR1-N1 plus mRNA was up-regulated (ANOVA F_6,14 _= 87.7, p < 0.0001) at 14 (p < 0.01), 21 (p < 0.01) and 28 days (p < 0.01) following TNBS treatment (Fig. 2C).

There was no apparent regulation of NR1 Exon 22 subunit after TNBS treatment. The mRNA level was similar to untreated and saline control rats (Fig. 2D).

Based on the RT-PCR results, we chose one time point, which was 14 days after TNBS induced colitis, to examine the functional protein expression during the colitis as well as to detect location of the NMDA receptor in the inflamed colon.

### Two-dimensional (2-D) gel analysis

The eight splice variants of NR1 are predicted to have different isoelectric points and slightly different molecular weights. We have adopted 2-D polyacryamide gel electrophoresis to detect the eight splice variants of the NR1 subunit in the colon.

**Figure 3:**
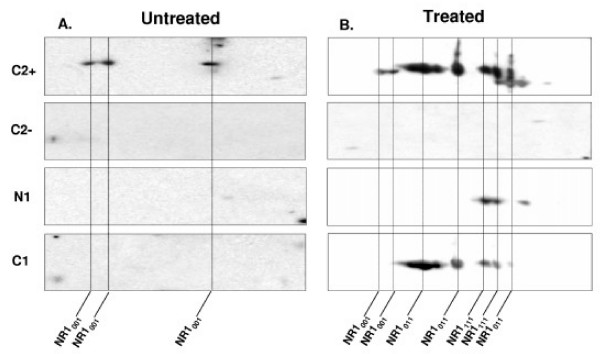
(A) 2-D western analysis of NR1 splice variants in rat's myenteric plexus. Fig. 3A: 2D gel analysis in normal control rat. (B) 2-D gel analysis 14 days following TNBS injection. To identify proteins with antibodies to the NR1-C2 plus, NR1-C2 minus, NR1-C1 and NR1-N1 in descending colon two separate membranes were used for all 4 experiments in the treated and control animals. The membranes were stripped of primary antibody between experiments. Label at the bottom indicated the NR1 splice variants.

In the untreated control group, only one subunit of NR1 receptor was expressed NR1_001 _(Fig. 3A) [39]. However, we found changes in protein profiles of NR1 subunits following TNBS treatment in rats (Fig. 3B). Among the eight potential splice variants of NR1 protein 3 subunits are observed following 14 days TNBS injection. They are NR1 _001_, NR1_011_, and NR1_111 _(Fig. 3B) [39].

### Immunocytochemistry

**Figure 4:**
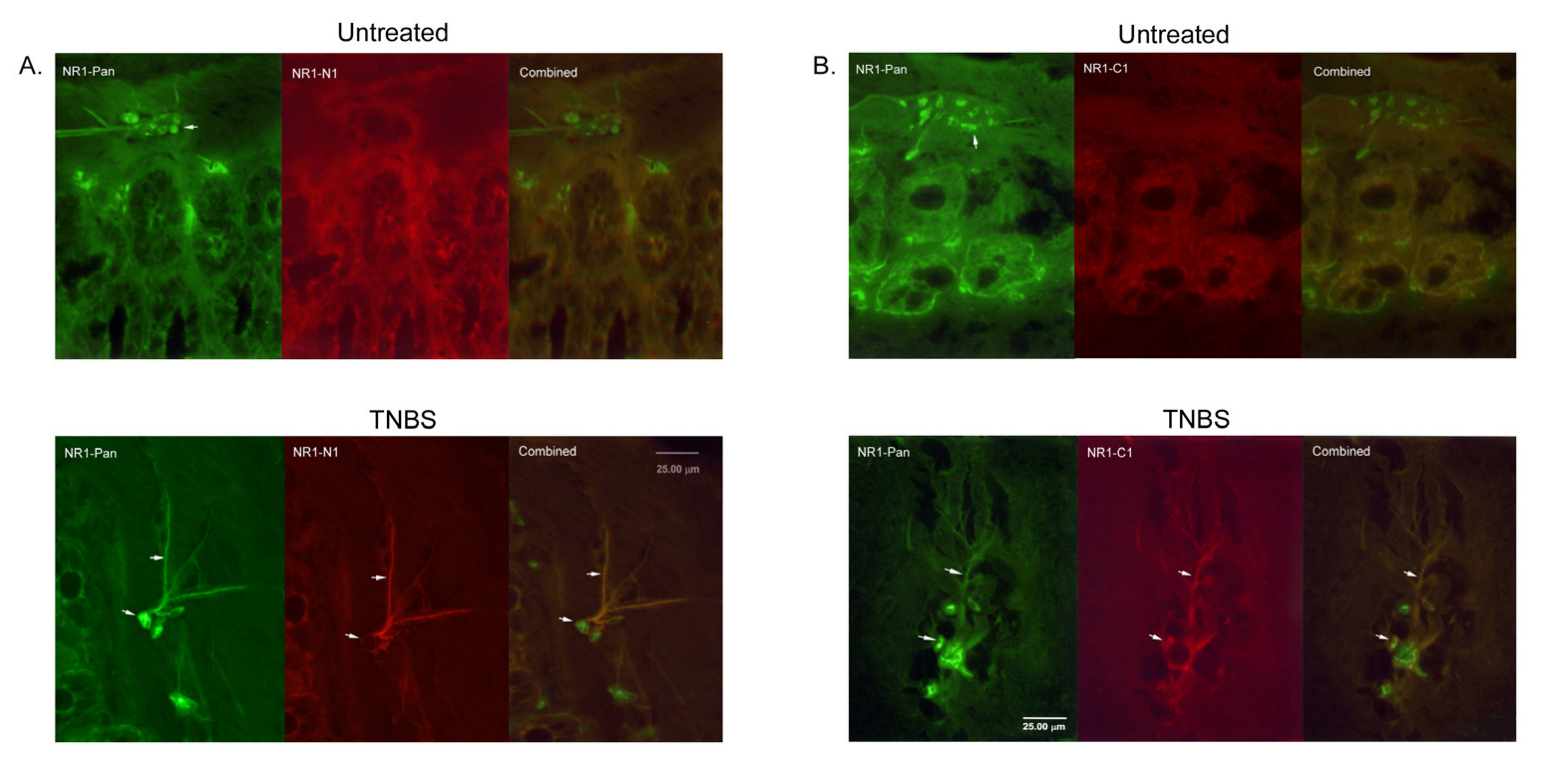
(A) Photomicrographs illustrating NR1 pan and NR1 N1 receptor double-labeling in descending colon. The upper panel is normal control rats. The lower panel is from inflamed rats. (B) Photomicrographs illustrates NR1 pan and NR1 C1 receptor double-labeling in myenteric plexus of descending colon. The upper panel is normal control rats. The lower panel is from inflamed rat.

Double-labeling fluorescent immunocytochemistry showed that the NR1-C1 and NR1-N1 are not expressed in the untreated control rat's colon (Fig. 4A and 4B). NR1 pan is present in enteric neurons in both untreated control rats and TNBS treated rats (see Fig 4A and 4B). Importantly, NR1-N1 appeared in TNBS induced colitis. Double-labeling of fluorescent immunocytochemistry for NR1 Pan and NR1-N1 indicated that they are located in neurons in the myenteric plexus of colon (Fig 4A) at 14 days following TNBS treatment. NR1 pan with NR1-C1 double-labeling was also present in neurons in the myenteric plexus (Fig. 4B) 14 days following TNBS treatment. The NR1-N1 and NR1-C1 only appeared in the myentric plexus (Fig. 4A and 4B).

## Discussion

This study characterized changes in NR1 expression in the colonic myenteric plexus in response to an inflammatory injury to the colon. The changes in enteric NMDA receptors is a novel finding in this study as previous studies have described NMDA changes in the spinal cord in response to a peripheral injury such as carrigenan or Complete Freund's Adjuvant (CFA) injection of the paw [20,51-53]. These changes in the NMDA receptor subunits in response to inflammation may have profound implications and could be involved in the pathophysiology of chronic visceral hypersensitivity seen in patients with post-infectious IBS and other chronic visceral pain disorders [54,55]. The current study examined the expression of the NR1 splice variants in the colon of rats following TNBS induced colitis. We found that protein expression of NR1_001, _NR1_011 _and NR1_111 _appeared in TNBS treated rats with active colitis. Untreated control rats only expressed NR1_001 _(Fig 3A). In addition, NR1-N1 and NR1-C1 protein expression was also present in the colonic myenteric plexus in TNBS treated rats. Parallel to the protein expression, the mRNA of NR1-N1 plus and NR1-C1 were also present at 14, 21 and 28 days after TNBS treatment.

The NR1 subunit forms eight functional splice variants based on the presence or absence of three alternatively spliced exons: Exon 5 (N1), Exon 21 (C1), and Exon 22 (C2). Splicing out the exon segment that encodes the C2 cassette removes the first stop codon, resulting in a new open reading frame that encodes an unrelated sequence of 22 amino acids (C2 minus) before a second stop codon is reached [35-39]. The NR1_001 _has Exon 5 and Exon 21 spliced out, while the Exon 22 is spliced in; NR1_011 _has the Exon 5 (N terminal) spliced out, while the Exon 21 and Exon 22 (C terminal) are spliced in; NR1 _111 _has all three exons [39,45].

The functional properties of NMDA receptors depend on the NR1 splice variant combination. NR1 receptors, lacking the N-terminal exon, exhibited a high affinity for NMDA and marked potentiation by spermine [45]. Presence of the N1 insert reduced the apparent affinity of homomeric NR1 receptors for NMDA and almost abolished potentiation by spermine at saturating glycine [45], while splicing-in the N1 insert increased current amplitude [36,41].

As demonstrated by Durand et al [45], NR1_001 _did not lead to generation of sufficiently large currents for analysis, even if the amount of RNA injected was increased from 10 to 50 ng per oocyte. In this study, we found NR1_001 _in the normal colon. Because Durand found that NR1_001 _does not generate significant current [45], NR1_001 _may not be part of a functional receptor. We have shown that NR1_011 _appeared only in rats following colonic inflammation. This has very important implications for visceral pain that NR1_011 _may play an important role in the plasticity that occurs following transient inflammation. NR1_111 _which has the N1 insert is also present in inflamed colon. Splicing in the N1 insert increased current amplitude [36,41]. Therefore NR1_111 _may increase the NMDA receptor activity in inflamed colon. In our immunocytochemistry study, the NR1-N1 also was expressed and presented on dendrites and cell membranes in the myenteric plexus following TNBS treatment (see arrow in Fig 4A). Expression of the N1 is associated with large current amplitudes and an enhanced responsiveness to PKC phosphorylation [39]. More importantly, NR1_011 _and NR1_111 _could modulate increased visceral hypersensitivity and alter colonic motility present in patients following transient inflammatory injury to the colon.

Phosphorylation of NMDA receptors is thought to be an important factor for cell modulation, regulation, and neuronal plasticity to response to a variety of stimuli. It may also play a critical role in long term potentiation (LTP) underlying memory formation. A number of residues that undergo phosphorylation are contained within a single alternatively spliced exon in the C-terminal domain, the C1 cassette [39,56]. Our immunocytochemistry revealed that the NR1-C1 protein was expressed on dendrites in the myenteric plexus (see arrow in Fig 4B) following TNBS treatment. The NR1-C1 was not expressed in the sub-mucosa. NR1-C1 contains an endoplasmic reticulum (ER) retention signal suggesting that the presence of C1 may alter translocation of NMDA receptors. The ER works as a control center in coordinating the sequential assembly of multi-subunit protein complexes within the ER and in defining the number of receptors expressed at the plasma membrane [57-61]. Scott et al [43] found that the ER regulates plasma membrane delivery of NMDA receptors. In addition, the study indicated that ER retention signals in the alternatively spliced C-terminal domain of the NR1 subunit control release of NR1 from ER and is regulated by PKC phosphorylation [43]. We hypothesize that TNBS induced colitis may produce assembled NR1 subunits and transports them through the ER-Golgi secretion pathway [62]. Phosphorylation blocks the NMDA receptor ER retention signal leading to surface expression. Our findings indicate that the activity of NMDA NR1 could be an important factor in the neuroplasticity that occurs in the colonic myenteric plexus following an inflammatory stimulus.

As might be expected during an acute inflammatory injury, there was visceral hypersensitivity present two days after TNBS injection that persisted to 28 days. However, the NMDA NR1 receptor up-regulation was present at 14–28 days following TNBS injection. The delay in NR1 expression could suggest that NMDA receptor may be involved in chronic nociception and may well persist following resolution of the colitis. Thus, it may take up to 14 days before more chronic changes occur, such as NMDA NR1 receptor up-regulation. A study of TNBS-induced colitis in rats [63] found hypersensitivity to visceral stimulation at 2 days after TNBS treatment, yet hypersensitivity to somatic stimuli was only present between 14 and 28 days. Interestingly, this time period is the same as the one wherein splice variants exhibited increased transcription in the present study. Although NMDA NR1 receptor up-regulation is not necessary for visceral hypersensitivity before 14 days, it may play a role in its maintenance at later stages when somatic hypersensivity develops. NMDA receptor upregulation is likely to be among one of multiple factors involved in the expression of visceral hypersensitivity.

Novel NR1 protein expression may be associated with persistent increases in impulse activity originating from the colon and rectum. This impulse activity could be generated as a result of increased NMDA receptor activity in the myenteric plexus and/or terminals of primary afferent neurons of the colon and rectum. The latter are known to have NMDA receptors [22]. Increased activity in myenteric neuronal NMDA receptors could lead to functional changes that stimulate receptors in the rectum and colon or increased NMDA receptors in primary afferent terminals could directly lead to their increased impulse activity. Regardless of the exact mechanism, increased impulse activity in afferents innervating the colon and rectum may then be transmitted to dorsal horn neurons of the spinal cord. The chronicity of the tonic afferent input from the viscera to the spinal cord may then lead to sensitization of dorsal horn nociceptive neurons and would be associated with visceral hypersensitivity. Somatic hypersensitivity could develop later as a consequence of long term tonic impulse activity and convergence of visceral and somatic primary afferent impulse inputs onto the same dorsal horn nociceptive neurons. In other words, somatic hypersensitivity would develop over time as a result of increased sensitization of somatovisceral convergent neurons

Spinal NMDA receptors are important in the induction and maintenance of central sensitization, yet peripheral NMDA receptors may also play an important role. Even when the peripheral inflammatory injury to the colonic myenteric plexus is healed, enduring neuroplastic changes in enteric neurons of the colon/rectum, and/or primary afferent terminals of the colon/rectum could lead to a condition of increased rectal and colonic hypersensitivity, as in irritable bowel syndrome. Similar to NMDA receptors in other tissues, NMDA receptor expression in the colonic myenteric plexus may be a major underlying factor in enhanced peripheral sensitivity in the rectum and colon. This and other peripheral factors may operate in concert with central sensitization mechanisms to produce visceral hyperalgesia and secondary somatic hyperalgesia.

In our previous study as well as in this study, there was persistent colitis up to 28 days following TNBS administration [63]. These findings differ from Asfaha et al. [64] in which the inflammation peaked at day 3 and resolved at week 6. Our findings may be different for several reasons. Asfaha et al. [64] used Wistar rats, whereas we used Sprague-Dawley rats. Secondly, they examined different time points which only included 3 days and then 6 weeks postinflammaion. They did not examine any timepoints inbetween to determine if there was persistent colitis (i.e. 7, 14, 21 and 28 days following TNBS injection). In addition, our study focused exclusively on neuronal plasticity of NMDA receptor expression following colitis. Finally, the intensity and duration of inflammation may very well be strain related. The study by Wells et al. [65] indicated that on days 2 and 4 post-TNBS, an overtly inflamed colon was present, frequently with adhesions; but by days 16 and 36, the acute effects of inflammation had resolved, but previously involved areas could be identified by mild adhesions and bowel wall thickening by using independent criteria of weight loss, histological evaluation of transmural inflammation and MPO (myeloperoxidase) analysis. Microscopic evaluation of TNBS-induced inflammation resulted in a transient increase in damage score with maximal inflammation present by days 4 post-TNBS and full resolution by days 36. Our data showed colonic inflammation present at day 2 through day 28 following TNBS treatment, but somatic hypersensitivity appeared from days 14 throughout to days 28. Several possibilities for these differences exist including differing concentrations of TNBS used and/or differing rat's age and/or weight may be reasons that the colonic inflammation healed early. Our data was most consistent with Morris' group's findings [46]. Their data indicated that the animals that received varying doses of TNBS in 50% ethanol developed areas of grossly visible bowel wall thickening, inflammation, and ulcers. These inflammation and ulcers were observed up to 8 weeks after administration of TNBS/ethanol.

There is increasing evidence indicating an important role for the NMDA receptor in mediating nociception in colon. The up-regulation of NMDA receptors was also shown in the spinal cord following colonic inflammation [7,66] suggesting central sensitization. This central sensitization may explain the somatic hypersensitivity our laboratory has found in both animals and humans with IBS [63,67,68].

## Conclusion

In summary, our current study identified the inflammation-induced changes in expression of individual colonic-NMDA receptor subunits. NR1 expression may play an important role in mediating visceral hypersensitivity in the TNBS colitis rat model. This could have important implications in the treatment of patients with chronic visceral pain disorders as NMDA receptor antagonists specifically targeting NR1 splice variants may be developed to control visceral pain. Future studies are needed to determine if NR1 expression continues to be increased following resolution of colitis.
